# Microbiological and Salivary Biomarkers Successfully Predict Site-Specific and Whole-Mouth Outcomes of Nonsurgical Periodontal Treatment

**DOI:** 10.3390/jcm13144256

**Published:** 2024-07-21

**Authors:** Ali JB Al-Sharqi, Ali Abdulkareem

**Affiliations:** Department of Periodontics, College of Dentistry, University of Baghdad, Bab Al Mudam, Baghdad P.O. Box 1417, Iraq; alijaafer@codental.uobaghdad.edu.iq

**Keywords:** periodontitis, nonsurgical periodontal treatment, periodontal pathogens, salivary biomarker, matrix metalloproteinase-9, glutathione S-transferase, Annexin-1

## Abstract

**Background/Objectives**: Nonsurgical periodontal treatment (NSPT) is the gold-standard technique for treating periodontitis. However, an individual’s susceptibility or the inadequate removal of subgingival biofilms could lead to unfavorable responses to NSPT. This study aimed to assess the potential of salivary and microbiological biomarkers in predicting the site-specific and whole-mouth outcomes of NSPT. **Methods**: A total of 68 periodontitis patients exhibiting 1111 periodontal pockets 4 to 6 mm in depth completed the active phase of periodontal treatment. Clinical periodontal parameters, saliva, and subgingival biofilm samples were collected from each patient at baseline and three months after NSPT. A quantitative PCR assay was used to detect the presence of *Fusobaterium nucleatum* and *Porphyromonas gingivalis* in the biofilm samples. Salivary biomarkers including matrix metalloproteinase (MMP)-9, glutathione S-transferase (GST), and Annexin-1 were assayed both qualitatively (Western blot analysis) and quantitively (ELISA). **Results**: NSPT yielded significant improvements in all clinical parameters, including a reduction in bacterial load and decreased levels of MMP-9 together with increased concentrations of GST and Annexin-1. The binary logistic regression suggested that the overall accuracy of *P. gingivalis* identification, probing pocket depth, and interproximal sites was 71.1% in predicting successful site-specific outcomes. The salivary biomarker model yielded an overall accuracy of 79.4% in predicting whole-mouth outcomes following NSPT. **Conclusions**: At baseline, the presence of shallow periodontal pockets at interdental locations with a lower abundance of *P. gingivalis* is predictive of a favorable response to NSPT at the site level. Decreased salivary MMP-9 associated with increased GST and Annexin-1 levels can predict successful whole-mouth outcomes following NSPT.

## 1. Introduction

The occurrence of periodontal disease begins with the accumulation of a dental biofilm, which eventually increases in mass, leading to incipient dysbiosis and gingivitis. If the biofilms remain undisturbed, frank dysbiosis may occur, resulting in chronic, non-resolving lesions and destructive inflammatory reactions (periodontitis) [1]. Buildup of the subgingival microbiome is a complex process involving gradual changes in structural composition, oxygen tension, pH, and types of nutrients, facilitating the appearance of pathobionts such as *Porphyromonas gingivalis*, which are responsible for periodontitis-associated destructive events [2,3]. *Fusobacterium nucleatum* is a bacterium that plays a key role in anaerobic shifting of the microenvironment, facilitating the growth of red complex pathogens [4]. These two closely connected periodontal bacteria are valid indicators of periodontitis severity and have been continuously investigated to support the diagnosis of periodontal disease and predict the outcomes of treatment [3].

Increased levels of inflammatory and proinflammatory cytokines in oral fluids such as saliva and gingival crevicular fluid (GCF) are one of the hallmarks of periodontitis, which is also associated with decreased concentrations of anti-inflammatory cytokines. Such an imbalance in the level of these molecules emerges from the persistent inflammation produced by the increased abundance of subgingival pathobionts in periodontal lesions [5]. Matrix metalloproteinase (MMP)-9 is one of the proteinases targeting type I collagen in periodontal ligaments, leading to the degradation of extracellular matrix proteins and the propagation of periodontitis [6]. Currently, MMP-9 available in oral fluids is considered a promising biomarker with potential applications in anticipating, diagnosing, and predicting the outcomes of periodontal disease [7,8,9]. Glutathione S-transferase (GST) is a detoxification enzyme that catalyzes a range of molecules, including environmental toxins and oxidative stress products [10]. The level of GST was found to significantly decrease in the whole saliva and gingival tissue samples of periodontitis patients when compared to the controls [11,12]. Moreover, the treatment of periodontitis patients with nonsurgical periodontal treatment (NSPT) significantly altered the concentration of this enzyme to a level comparable to that of controls [11]. Additionally, polymorphisms of the *glutathione* gene were reported to be closely associated with increased susceptibility to periodontitis [13]. Proteomic studies of the gingival tissues revealed the presence of Annexin-1, which is an anti-inflammatory protein that inhibits the accumulation of neutrophil in tissue [14,15]. Salivary Annexin-1 has emerged as a strong candidate biomarker for predicting and diagnosing periodontitis. In the offspring of periodontitis patients, Annexin-1 was 7.1 times less commonly produced than in the control counterparts [16]. Proteomic analysis has consistently detected 5-fold higher Annexin-1 levels in the GCF of periodontally healthy subjects [17]. Additionally, this salivary molecule has shown diagnostic potential in differentiating gingivitis in pregnant women [18]. The number of clinical studies investigating the role of GST and Annexin-1 in predicting the outcomes of NSPT remains limited, and the majority of published studies on this biomarker are observational.

The most common treatment for periodontal disease is centered around reducing pathogenic subgingival dental plaque biofilm. NSPT is the recommended technique used to treat shallow to moderately deep periodontal pockets and reduce the bacterial load from deep periodontal pockets before the surgical phase [19]. Both manual instruments and ultrasonic scalers are equally effective in performing NSPT [19]. The reduction in putative periodontal pathogens following NSPT was also reported by several studies [20,21,22]. Belstrøm et al. demonstrated a relatively significant increase in the abundance of beneficial bacteria due to the reduction in pathogenic bacteria in the subgingival microbiota after periodontal treatment [23]. However, unfavorable outcomes of NSPT are expected in some cases due to the failure of eliminating pathogenic bacteria and restoring the symbiotic state of the subgingival microbiota [24,25,26]. Several local and systemic factors could modulate responses to NSPT, together with an individual’s genetic susceptibility to periodontitis [24,27]. Therefore, adjunct treatments are often combined with periodontal therapy to increase the odds of success [28].

The use of biomarkers available in oral fluids to diagnose periodontal disease and predict the outcomes of periodontal therapy has become a key area of study in the literature published in recent decades [8,29]. Therefore, this study was conducted to determine the predictive potential (whole-mouth and site-specific) of selected host-derived bacterial biomarkers for the outcomes of NSPT.

## 2. Materials and Methods

### 2.1. Study Design

This prospective interventional cohort was conducted on patients seeking treatment for periodontitis at the College of Dentistry, University of Baghdad, between January 2023 and February 2024. All patients were recruited consecutively and treated in accordance with the ethical rules outlined by the World Medical Association Declaration of Helsinki and its later amendments. This study was approved by the Ethics Committee, College of Dentistry, University of Baghdad (Ref. # 663, project # 663622, Date 13 September 2022). A signed consent form was obtained from each patient after being fully informed about the details of this cohort study.

### 2.2. Inclusion/Exclusion Criteria

The recruited patients were adults (≥18 years) who did not report any history of systemic diseases/conditions. All patients were diagnosed with periodontitis of any stage and grade, which was defined by detectable interdental clinical loss of attachment (CAL) affecting two or more non-adjacent teeth. Periodontitis was also confirmed when facial/oral surfaces exhibited CAL ≥ 3 mm together with probing pocket depths (PPD) ≥ 4 mm in 2 or more non-adjacent teeth [30]. The extent of periodontitis in all cases was generalized, with CAL affecting >30% of teeth. We also included cases with moderately deep pockets (4–6 mm) without furcation involvement and that were intended to be treated with NSPT. Smokers, those with systemic diseases such as diabetes mellitus, those not diagnosed with periodontitis, those with periodontal pockets > 6 mm, pregnant individuals, patients scheduled for surgical interventions, those enrolled in other active studies or treatment programs, and those not showing a desire to participate were also excluded from the study.

### 2.3. Clinical Parameters and Intervention

At baseline, all patients received full-mouth periodontal charting, which included recording the plaque index (PI) [31], bleeding on probing (BOP) [32], PPD, CAL, and number of missing teeth. All these parameters were recorded using an UNC-15 periodontal probe (Medesy, Maniago, Italy), with 6 sites per tooth, except for PI, which was recorded by the same calibrated periodontist at 4 surfaces (A.J.A.). PI was recorded using a disclosing agent (Biofilm Disclosure, EMS, Nyon, Switzerland). Intra-examiner consistency was conducted before the start of the study and repeated once every 3 months. The accepted level of consistency was >80% for categorical variables (PI and BOP) using the kappa-coefficient test and >90% for continuous parameters (PPD and CAL), as determined with an interclass coefficient test. After recording the parameters and confirming the diagnosis using radiographs, the cases were subclassified according to stage and grade based on criteria recommended by the American Academy of Periodontology (AAP)/European Federation of Periodontology (EFP) joint workshop [30].

At the baseline visit, salivary samples and subgingival biofilm were first collected. Then, treatment was delivered following the EFP guidelines for treating periodontitis [19]. After diagnosis and assessing the absence of risk factors, each patient received proper oral hygiene instructions with supragingival and subgingival professional mechanical plaque removal (PMPR) using an ultrasonic scaler (Woodpecker, Ultrasonic Piezoelectric Scaler UDS-A, Guilin, China). One week later, root surface debridement (RSD) was conducted for all periodontal pockets following a 24 h protocol [33] using Gracey curettes (Medesy, Italy) under local anesthesia. All patients were supplied with the same type of toothbrush, toothpaste, and interdental brush (CURAPROX, Kriens, Switzerland). The appropriate diameter of the interdental brush used by each patient was determined using an interdental access probe provided with the kit to measure the patient’s interdental spaces. The patients were instructed to return after 3 months for re-evaluation. During this visit, the same examiner collected salivary and biofilm samples and re-recorded the relevant clinical parameters. At the endpoint of the study, treatment was re-evaluated, and patients exhibiting residual pockets (PPD ≥ 4 mm with BOP) were referred for the corrective phase [19].

### 2.4. Outcomes

The primary outcome of this study was achieving pocket closure, which was defined according to the site, tooth, and whole oral cavity levels. For each site, a successful outcome at 3 months following termination of the active phase of periodontal therapy was defined as PPD ≤ 4 mm with no BOP [34]. The treatment was considered a failure when sites with PPD ≤ 4 mm exhibited bleeding tendencies or the presence of residual pockets > 5 mm with or without BOP. The same criteria were applied to the tooth level, for which any pockets detected in each tooth were required to fulfill the above-mentioned criteria at the endpoint of the study. For the whole mouth, a successful outcome was defined as a mean PPD ≥ 1.29 mm together with a mean CAL gain ≥ 0.55 mm [35]. All other clinical parameters, including PI, BOP, and CAL, together with microbiological and biomarker levels, were considered to be secondary outcomes.

### 2.5. Sample Size

A priori sample size calculation was based on the primary outcome of pocket closure. This value was obtained from a previous study that reported a ratio of 1.6 for successful/unsuccessful treatment [36]. This analysis was performed using a chi-square test with the G*Power software (version 3.1.9.7) for Windows (Universität Düsseldorf, Düsseldorf, Germany), setting the α error probability as 5% with 80% power. The results showed that a sample size of 68 patients was sufficient to reject the null hypothesis. This value was further increased to 75 patients by adding 10% of the calculated sample to avoid dropout for any reason.

### 2.6. Subgingival Biofilm Sampling

Subgingival plaque sampling was conducted as previously described [37]. After carefully removing any supragingival calculus/deposits, the selected sites were isolated with cotton rolls and gently dried with an air stream. Then, a sterile Gracey curette was carefully inserted into the most apical extension of the periodontal pocket, pulled coronally, and moved along the root surface in a single stroke with constant pressure. The curette tip containing the subgingival biofilm sample was immediately placed and gently shaken to suspend the plaque sample in an Eppendorf microcentrifuge tube (1.5 mL). This tube contained 500 μL TE buffer (10 mM Tris-HCl and 1 mM EDTA; pH 8.0) as a suspended solution. The tubes were centrifuged (Microfuge IB Centrifuge, Beckman Coulter, Krefeld, Germany) at 8000× *g* for 8 min. The formed pellet was washed with 500 μL TE buffer (x5), and samples were stored at −20 °C until DNA extraction. All sampling procedures were performed by a single operator throughout the study.

### 2.7. Unstimulated Saliva Collection

The passive drooling technique was used in this study to collect unstimulated whole saliva samples [8]. At the baseline visit, the patients were asked to refrain from eating or drinking for 1 h prior to sampling. Each patient was asked to sit upright with their head tilted forward and downward to allow saliva to pool in front of the mouth. The saliva was then allowed to passively collect in a polypropylene cup. This process was terminated when bubbling of the saliva was observed. The salivary sample was aspirated into a plastic Eppendorf tube containing 50 μL protease inhibitor solution and then centrifuged at 4000× *g* for 3 min to remove debris. The resulting supernatant was stored at −20 °C to subsequently measure the levels of biomarker proteins. All samples remained frozen for a maximum of 1 month before being analyzed.

### 2.8. Real-Time PCR Assay for F. nucleatum and P. gingivalis

Lysis of the bacterial pellet was performed using G-spin Genomic DNA Extraction (Intron, Gyeonggi-do, Korea). The sequence of the forward primer for *F. nucleatum* was 5′-GGATTTATTGGGCGTAAAGC-3′, that of the reverse primer was 5′-GGCATTCCTACAAATATCTACGAA-3′, and that of the Taqman probe was 5′-CTCTACACTTGTAGTTCCG-3′. The sequence of the forward primer for *P. gingivalis* was 5′-TGCAACTTGCCTTACAGAGGG-3′, that of the reverse primer was 5′-ACTCGTATCGCCCGTTATTC-3′, and that of the Taqman probe was 5′-AGCTGTAAGATAGGCATGCGTCCCATTAGCTA-3′. All primers and TaqMan probes were purchased from the Macrogen company (Seoul, Korea). PCR amplification was conducted in a total reaction mixture volume of 20 μL. This mixture consisted of the forward primer, reverse primer, and probe (concentration, 10 µM/µL; volume, 1 µL each) added to 5 µL of double-deionized water, 10 µL of GoTaq^®^ Probe qPCR Master Mix (Promega, Madison, WI, USA), and 2 µL of purified DNA from the plaque samples. The microplates were then positioned in a thermal cycler (Stratagene, La Jolla, Ca, USA) and heated for 10 min at 95 °C, followed by 40 cycles of 95 °C for 15 s and 60 °C for 1 min. The amplifying success rate was greater than 95%, with a calculated error rate based on PCR duplicates of less than 1%. Standard curves previously prepared were used to calculate the *C_t_* values for each bacterium.

### 2.9. Assaying Salivary Proteins

Salivary proteins were assayed using two techniques: a Western blotting assay (WBA) and enzyme-linked immunosorbent assay (ELISA). For the WBA, a RIPA lysis buffer (E-BC-R327, Elabscience®, Houston, TX, USA) was used to extract protein samples, which were then separated via 10% SDS-PAGE. This procedure was followed by transferring the samples onto a polyvinylidene difluoride (PVDF) membrane that was blocked with 5% bovine serum albumin (BSA) for 1.5 h at 25 °C. Then, the membranes were incubated at 4 °C overnight with primary antibodies purchased from Elabscience^®^, including monoclonal MMP-9, polyclonal GSH, and polyclonal Annexin-1, all at a dilution of 1:1000. This process was followed by incubating the membranes with a secondary antibody conjugated with horseradish peroxidase (1:5000, Elabscience^®^) for 1 h at room temperature using a chemiluminescent substrate detection system (Elabscience^®^, China). PVDF membranes were loaded into the X-ray cassette, working solution was added, and the exposure time was adjusted to capture the best image.

Commercially available ELISA kits for MMP-9, GST, and Annexin-1 were used to quantify the levels of these biomarkers in the saliva samples. All kits were purchased from Elabscience^®^ (Houston, TX, USA) and based on the sandwich technique. The technical procedures were followed as instructed by the manufacturer for each kit. A wavelength of 450 nm was used to measure the optical density (OD) for MMP-9, GST, and Annexin-1. The OD values were converted into corresponding concentrations using the regression formula obtained from the standard curve for each protein. All experiments were run in duplicate, and the readings were averaged based on the standard curve and samples.

### 2.10. Statistical Analysis

The results were expressed as the mean, standard deviation, and standard error for continuous data, while frequency and percentage were used for categorical variables. The normal distribution of data was assessed using the Shapiro–Wilk test, which indicated that the clinical parameters were parametric, whereas the microbiological and biochemical data were non-parametric. Accordingly, differences between the baseline and endpoint of the study were analyzed using paired *t*-tests for clinical parameters and Wilcoxon’s ranked assay for microbiological and biochemical variables. A chi-square test was used to assess the differences between frequencies of the categorical variables. Binary logistic regression models were used to determine the potential of different variables to predict the outcomes of NSPT. The success/failure of treatment was dichotomized into 0 and 1 based on previously determined thresholds at the whole-mouth and site levels. Demographic, clinical, microbiological, and salivary proteins were included as predictors of outcomes. Accuracy and cut-off points for these predictors were determined using area under the curve (AUC) and receiver operating characteristic (ROC) analyses. The difference level was considered significant when the *p*-value was less than 5%. Statistical analyses were performed using the GraphPad Prism (version 9.0, GraphPad Prism Software, Boston, MA, USA) and SPSS (version 26, IBM, Chicago, IL, USA) software.

## 3. Results

### 3.1. Basic Demographic and Clinical Variables

During the recruitment stage, 274 periodontitis patients were screened for their eligibility to be included in this interventional cohort. A total of 72 patients met the inclusion criteria and agreed to participate. However, four were excluded from final analysis due to a lack of compliance with the scheduled appointments. The final sample consisted of 68 patients (31 male and 37 female) with an average age of 55.4 ± 6.3 years (Table 1). Table 1 illustrates the distribution of these patients according to the stage and grade of periodontitis together with the average number of missing teeth. The total number of moderately deep periodontal pockets (4 to 6 mm) was 1111, which were considered to indicate disease activity either through an increase in depth (≥5 mm) with no BOP (n = 692) or PPD ≥ 4 mm associated with BOP (n = 419) (Table 1).

### 3.2. Clinical Outcomes

Table 2 illustrates the clinical outcomes after NSPT. Three months following the termination of active NSPT, all clinical parameters, including PI, BOP, PPD, and CAL, showed significant improvements compared to the baseline. Additionally, residual periodontal pockets (≥5 mm without BOP) represented 10.8% of the total pockets, while 18.1% of the periodontal pockets ≥ 4 mm exhibited BOP. The number of patients who presented successful outcomes at the whole-mouth level was 42 (61.8%), while the rest failed to meet the targeted success level. At the tooth level, the overall success rate was about 70% for all teeth, with maxillary teeth showing more favorable responses to NSPT than mandibular teeth. Out of the 1111 sites treated with NSPT, complete pocket closure was observed at 790 (71.1%) sites.

**Figure 1:**
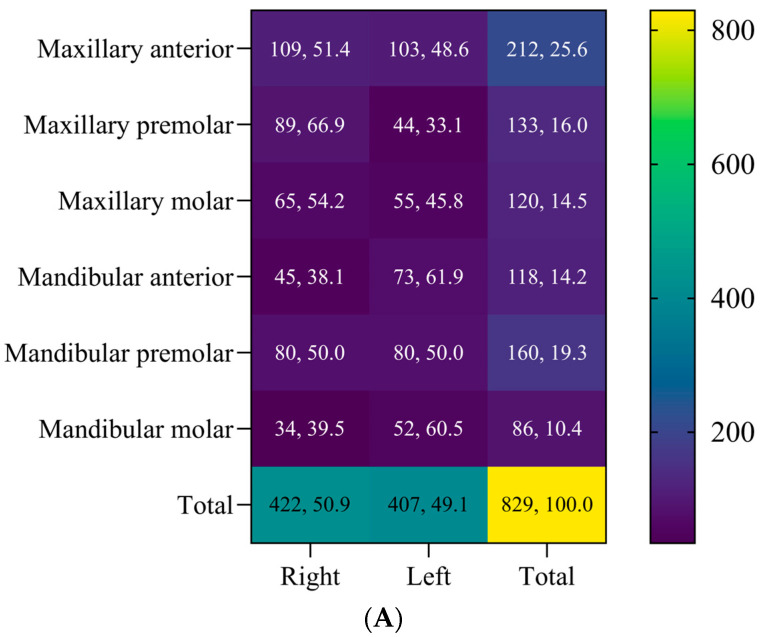

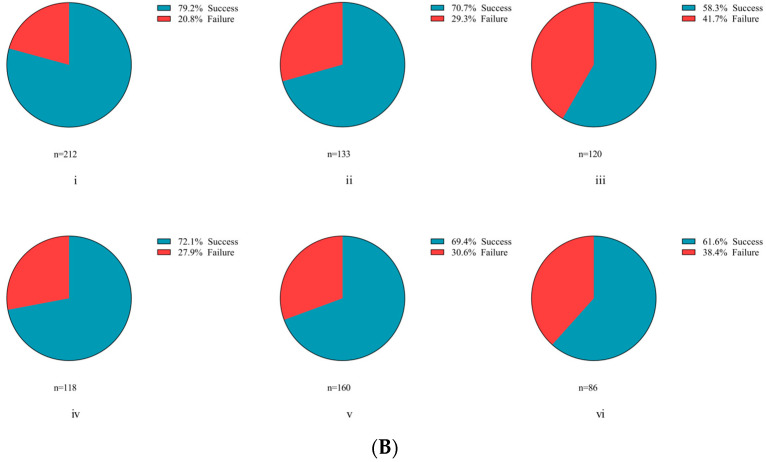
Distribution of pockets according to tooth type (**A**) and outcomes of nonsurgical periodontal therapy (NSPT) (**B**). A total of 829 teeth exhibiting a probing pocket depth of 4 to 6 mm were included. The majority of periodontal pockets were detected in the maxillary anterior teeth (n = 212, 25.6%), while mandibular molars showed the lowest frequency (n = 86, 10.4%). Outcomes of NSPT were determined for the maxillary anterior teeth (**i**), maxillary premolars (**ii**), maxillary molars (**iii**), mandibular anterior teeth (**iv**), mandibular premolars (**v**), and mandibular molars (**vi**). The highest success rate was observed in the maxillary anterior teeth (79.2%), while maxillary molars yielded the lowest success rate (58.3%).

The distribution of periodontal pockets according to teeth (Figure 1) showed the maxillary anterior teeth to be the most affected (n = 212, 25.6%), whereas the least affected teeth were the mandibular molars (n = 86, 10.4%). Successful pocket closure was greatest for the maxillary anterior teeth (79.2%), followed by the mandibular anterior teeth (72.1%), maxillary premolars (70.7%), mandibular premolars (69.4%), mandibular molars (61.6%), and maxillary molars (58.3%) (Figure 1).

**Figure 2:**
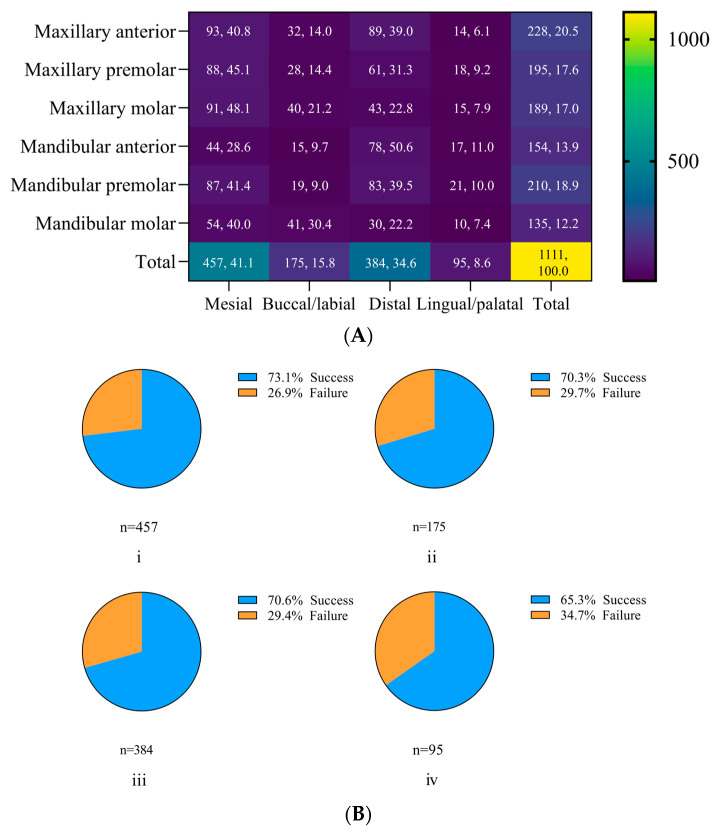
Distribution of pockets according to site (**A**) and outcomes of nonsurgical periodontal therapy (NSPT) (**B**). A total of 1111 sites exhibiting probing pocket depth of 4 to 6 mm were included. The majority of periodontal pockets were detected at mesial sites, followed by distal, facial, and oral surfaces. The highest success rate was observed on (**i**) mesial surfaces (73.1%), followed by both distal (**iii**) and facial (**ii**) sites (about 70%) and (**iv**) oral surfaces (65.3%).

Out of the 1111 sites, the highest frequency of periodontal pockets was observed on the mesial surfaces of the teeth, followed by the distal, facial, and oral surfaces (Figure 2). Following NSPT, the highest success rate in pocket closure was observed on the mesial surfaces (73.1%), followed by the distal (70.6%), facial (70.3%), and oral surfaces (65.3%) (Figure 2).

### 3.3. Microbiological Outcomes

**Figure 3:**
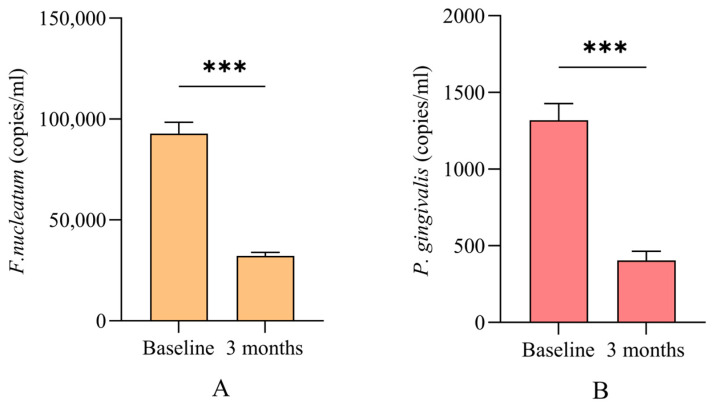
Number of *Fusobacterium nucleatum* (**A**) and *Porphyromonas gingivalis* (**B**) DNA copies in subgingival biofilm before and after nonsurgical periodontal therapy (NSPT). The number of DNA copies for both bacteria significantly decreased following NSPT. *** significant difference at *p* < 0.001 using Wilcoxon’s ranked assay. Data are presented as the mean ± standard error.

At baseline, the positive presence of *P. gingivalis* and *F. nucleatum* was detected at 22.5% and 94.8% of the sites, respectively. The abundance of these bacteria decreased significantly to 16.8% for *P. gingivalis* and 47.2% for *F. nucleatum* at 3 months following NSPT. Sites that did not favorably respond to NSPT demonstrated the presence of *P. gingivalis* and *F. nucleatum* at 38.5% and 27.1% of the sites, respectively. Both bacteria were positively expressed in 30.5% of these sites (Table 3). *P. gingivalis* and *F. nucleatum* were quantitatively measured using PCR (Figure 3). The number of DNA copies for both bacteria significantly decreased 3 months after NSPT (Figure 3).

### 3.4. Levels of Salivary Proteins

**Figure 4:**
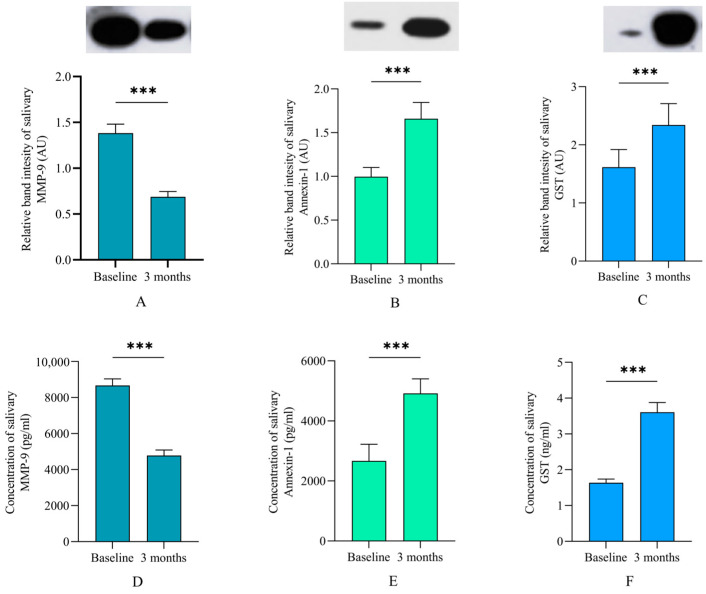
Levels of salivary proteins at baseline and 3 months after nonsurgical periodontal therapy (NSPT). Western blot analysis (**A**–**C**) showed that the matrix metalloproteinase (MMP)-9 level significantly decreased 3 months after NSPT, while levels of annexin and glutathione S-transferase (GST) increased during the same period. The same pattern was observed when using ELISA (**D**–**F**) to assay the concentrations of these proteins. *** A significant difference at *p* < 0.001 using Wilcoxon’s ranked assay. Data are presented as the mean ± standard error.

Levels of salivary proteins including MMP-9, GST, and Annexin-1 were assayed both qualitatively (WBA) and quantitatively (ELISA) (Figure 4). The WBA results showed that both techniques significantly decreased the levels of salivary MMP-9 three months after the termination of NSPT compared to the baseline levels. This result was associated with a significant increase in the levels of salivary GST and Annexin-1 during the same period.

### 3.5. Predictive Potential of Salivary and Bacterial Biomarkers for NSPT

The potential of using salivary and microbiological biomarkers to predict outcomes of NSPT was determined using binary logistic regression models (Table 4). Subsequent analysis showed that an increased *P. gingivalis* count and PPD at baseline could be used as site-specific predictors for the failure of NSPT. This model also showed the distal site of the teeth to be positively associated with favorable outcomes, as the odds of pocket closure increased by 1.374 after NSPT. For salivary biomarkers, all included proteins (MMP-9, GST, and Annexin-1) showed a significant association with the outcomes of NSPT. An increased level of MMP-9 at baseline was associated with unfavorable outcomes, whereas high GST and Annexin-1 levels predicted a higher probability of pocket closure following NSPT.

**Figure 5:**
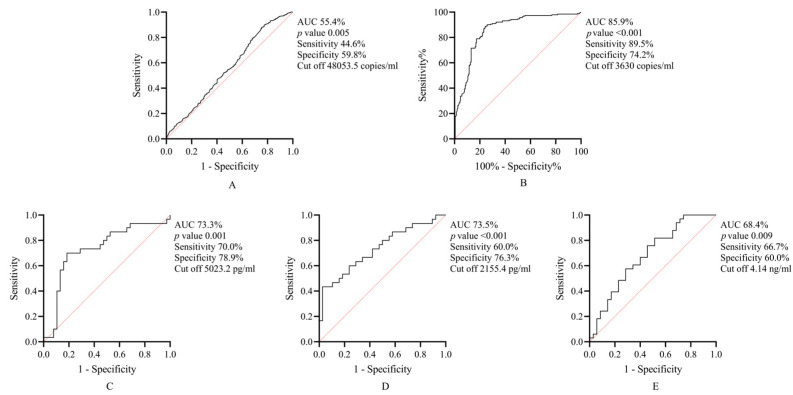
ROC curves of microbiological and salivary biomarkers. Both *Fusobacterium nucleatum* (**A**) and *Porphyromonas gingivalis* (**B**) showed potential in predicting site-specific outcomes of nonsurgical periodontal therapy (NSPT). However, *P. gingivalis* offered higher accuracy (AUC 85.9%) than *F. nucleatum* (AUC 55.4%). Similarly, whole-mouth outcomes after NSPT could be accurately predicted using salivary matrix metalloproteinase-9 (**C**), Annexin-1 (**D**), and glutathione S-transferase (**E**). Red dotted line represents line of equality.

ROC analysis was performed to identify the sensitivity, specificity, and cut-off points of salivary and microbiological biomarkers for predicting the outcomes of NSPT (Figure 5). The AUC of *P. gingivalis* was about 85%, with good sensitivity and specificity. The rate of successful pocket closure is expected to increase when the load of this bacteria is <3630 copies/mL in the periodontal pocket. Similarly, the *F. nucleatum* cut-off point to predict the outcomes of NSPT was found to be 48,053 copies/mL. However, *F. nucleatum* produced much lower AUC, sensitivity, and specificity values compared to *P. gingivalis*. For salivary biomarkers, successful outcomes could be predicted when the level of MMP-9 (AUC 73.3%) was less than 5023 pg/mL, whereas we considered increased levels of salivary Annexin-1 (AUC 73.55) and GST (AUC 68.4%) beyond 2155 pg/mL and 4.14 ng/mL, respectively, at baseline as predictors of success.

## 4. Discussion

The results of this interventional study demonstrated the potential of salivary and microbiological biomarkers in accurately predicting the outcomes of NSPT. This result applied to site-specific, *P. gingivalis*, and whole oral cavity levels (MMP-9, GST, and Annexin-1). The greatest level of pocket closure was observed on the maxillary anterior and proximal surfaces of the teeth, whereas failure of treatment was mostly associated with molar teeth and oral sites. The most common conventional technique for treating periodontal disease is mechanical debridement, which exhibits considerable efficacy in achieving pocket closure. However, the complete suppression of pathogenic biofilms using this technique is not always guaranteed, possibly leading to an unfavorable response and/or disease recurrence [38]. Clinically, the ability to predict NSPT outcomes is crucial and would greatly help medical professionals customize the treatment plan for each patient, thereby saving the time and costs associated with unnecessary and lengthy treatment.

According to the latest guidelines, the initial phases of periodontitis treatment involve controlling local and systemic risk factors, giving oral hygiene instructions, and behavioral modification followed by PMPR [19]. In these phases of treatment, combining supragingival plaque control with subgingival debridement is the cornerstone of successful outcomes [39]. The oral hygiene levels among the patients in this study showed substantial improvements due to the provision of meticulous oral hygiene instructions and compliance of the patients, as demonstrated by a significant reduction in PI scores 3 months after the termination of treatment. NSPT is usually advised to treat shallow to moderately deep periodontal pockets, with favorable outcomes expected in a majority of cases [40,41]. Success is defined by a PPD ≤ 4 mm with no BOP, but this is not always observed in clinical settings. A lack of positive results can be attributed to several factors, including a lack of/poor compliance with oral hygiene instructions, insufficient debridement, and individual susceptibility to the disease [24,27]. Other factors such as diabetes mellitus, smoking, teeth with deep periodontal pockets (≥6 mm), and posterior teeth with furcation are factors that substantially contribute to unfavorable outcomes following periodontal therapy [24,25,26]. However, these factors were excluded during the recruitment phase to avoid any possible biases in the results.

Reducing the bacterial load of Gram-negative anaerobes and disrupting biofilm structure are the prime goals of periodontal therapies. *P. gingivalis* is a “key” periodontal pathogen responsible for eliciting intense immune/inflammatory responses and extensive tissue destruction, even when present in low abundance [38]. Similar to the results from this study, the failure of NSPT, with or without adjunctive therapy, to reduce the load of periodontal pathogens such as *P. gingivalis* and *F. nucleatum* is a common finding among a range of clinical studies [38,42]. In certain cases, the subgingival microbiota can revert to their original composition before treatment, especially in the absence of appropriate oral hygiene. This outcome could be attributed to the intraoral distribution of periodontal pathogens able to colonize the whole oral cavity, including nondental locations such as the dorsum of the tongue and the tonsils, which provide reservoirs for these bacteria to recolonize recently treated sites [38,43,44]. Additionally, the topographic complexities of the root surface such as the presence of grooves, depressions, and gouges/scratches represent excellent sheltered areas that protect periodontal bacteria against mechanical debridement [24,45]. This result further highlights the importance of full mouth debridement and good oral hygiene [46,47,48]. However, the recolonization of periodontal pockets by pathogenic bacteria is ultimately dependent on the treatment protocol, type of periodontal pathogens, and oral hygiene measures [38].

According to a previous retrospective study, the highest failure rate of NSPT was reported in association with molars. Success is further compromised when the furcation area is involved, with increased success observed in association with the anterior teeth and premolars [40]. These results are consistent with a previous work showing a similar pattern of NSPT outcomes according to tooth type. Additionally, Chen et al. [49] indicated that increased favorable outcomes of NSPT occur in association with interproximal surfaces, which is also consistent with the results of the present study.

The qualitative and quantitative microbiological analyses indicated a significant reduction in the bacterial load of periodontal pathogens *F. nucleatum* and *P. gingivalis*, together with improved clinical outcomes. This result agrees with another study, which showed that NSPT for moderate periodontitis cases significantly decreased *P. gingivalis*, *Tannerella forsythia*, *F. nucleatum*, *Aggregatibacter actinomycetemcomitans*, and *Actinomyces* ssp. [21]. The relative reduction in *P. gingivalis* and *T. forsythia* was associated with a significant increase in symbiotic bacteria, including *Streptococcus*, *Rothia*, and *Actinomyces*, in the subgingival microbiota 3 months after periodontal therapy [23]. Treating periodontal pockets in patients with stage III/IV periodontitis using range of nonsurgical options significantly reduced the putative periodontal pathogens, including *P. gingivalis*, *Eubacterium nodatum*, and *Prevotella* ssp. [22]. However, the authors suggested that this improvement could be transient and may be followed by deterioration of the microbiome profiles 6 months after treatment. This result highlighted the importance of profiling subgingival microbiota to help customize individualized therapies [22].

The oral cavity is a rich source of biomarkers, which are available in biofluids such as saliva, GCF, and oral rinse samples, together with bacteria and their products in dental plaque biofilms [50]. The immune responses elicited in response to the presence of pathobionts in subgingival biofilm are accompanied by the release of molecules, including enzymes, tissue breakdown products, and cytokines [51,52]. All these molecules represent potential biomarkers that could be used to predict, diagnose, and monitor periodontal disease. Saliva is the most popular source of biomarkers in clinical studies due to its rapid and easy collection, abundance of proteins, and availability in relatively large volumes [50]. Therefore, saliva was selected in this study to analyze the relevant biomarkers.

Previous reports demonstrated that GST levels were significantly decreased in GCF, saliva, and serum samples from periodontitis patients compared to the levels in healthy individuals and increased following periodontal treatment [11,12]. This result agrees with the findings of the present study, which indicated restoration of the redox system to a healthy state. This outcome was further supported by results from another study, which confirmed the perturbation of intracellular GST in patients with periodontitis, leading to an aberrant neutrophil chemotaxis and compromising the patient’s ability to handle increased oxidative stress [53,54]. However, other studies presented opposite results [55,56,57], possibly due to differences in sample size, sampling procedures, stages of disease, and the oral fluid used for analysis. Increased MMP-9 levels are a distinct feature of progressive periodontitis and are mainly produced by neutrophils and macrophages being continually recruited to the periodontal lesion [58]. The concentration of MMP-9 was found to significantly decrease 3 months after NSPT [59], which is consistent with the present findings. Increased Annexin-1 levels after NSPT indicated that the redox system was restored to a normal state, which is in accordance with the anti-inflammatory role of this molecule [16,17].

The use of different variables to predict the increased severity of periodontitis and the outcomes of periodontal therapy has gained popularity in the last decade [60,61]. In a one-year follow-up study, Baelum and López [60] suggested that abundant subgingival calculus and suppuration could predict improvement and tooth loss. Similar to the results of the present study, Chen, Yin, Chang, Kao, Tu, and Chen [49] indicated that baseline PPD and the presence of pockets on interproximal surfaces could predict the outcomes of NSPT. The authors of the latter study observed the presence of wider radiographic angles at distal sites associated with shallow infrabony defects, explaining why these sites responded more favorably to NSPT. Additionally, successful pocket closure could be predicted at sites with PPD < 5 mm [62] due to their greater accessibility, thus improving the chances of debriding most of the dysbiotic subgingival microbiota. Likewise, the success rate in this work was associated with periodontal pockets at interproximal sites and baseline PPD < 6 mm. Results from several studies have demonstrated promising potentials of salivary biomarkers to diagnose/monitor periodontal disease and predict outcomes of periodontal treatment [63,64,65,66,67,68]. However, the level of evidence is insufficient to state a concrete conclusion about the use of these biomarkers for the above-mentioned aspects [65,67]. Further studies were recommended by these studies to validate a single or combination of biomarkers as chair-side point-of-care testing in clinical practice. The available literature suggests introducing salivary MMP-9 as an efficient biomarker for the diagnosis of periodontitis with a diagnostic ability > 80% [8,69]. The accuracy of MMP-9 in predicting and monitoring the level of inflammation of periodontal tissue after treatment was assessed in other studies [70,71], whose results have highlighted this enzyme as a promising prognostic biomarker. Reports investigating the predictive potential of GST and Annexin-1, however, remain limited. The present study showed that both molecules are functional candidates for predicting NSPT outcomes, as their levels significantly increased after treatment, confirming their association with periodontal health. Indeed, GST is a member of the enzymatic group of the antioxidant system responsible for neutralizing increased oxidative stress associated with periodontitis [72]. Annexin-1 is a potent endogenous anti-inflammatory molecule known to modulate leukocyte-mediated immune responses and restore one’s homeostatic state [73]. Annexin-1 has shown promising potential in the diagnosis, prediction, and monitoring of periodontitis [16,17,18]. However, few clinical trials have explored this biomarker, creating challenges in confirming its predictive power.

The use of periodontal pathogen taxa to predict the outcomes of NSPT was based on the notion that each inflammatory exacerbation episode associated with periodontal tissue destruction must be preceded by a dysbiotic shift of the subgingival microbiota [74,75]. Today, qPCR-based kits to identify microbial signatures are commercially available. Such kits can recognize 5 to 11 periodontal bacteria species to diagnose, monitor, and predict periodontal disease [3]. A previous clinical cohort determined that a level of ≥1 × 10^5^ *P. gingivalis* cells/mL in saliva was positively correlated with clinical parameters and *P. gingivalis* abundance in subgingival biofilm. The reported sensitivity, specificity, and accuracy of the PCR-based device in the latter study were 95.0%, 93.3%, and 94.0%, respectively [76]. Clinical success at the site level was determined with 18% sensitivity and 60% specificity 6 weeks after treatment based on the absence of *P. gingivalis* [77]. Additionally, inferior NSPT outcomes were reported when the percentage of *P. gingivalis*, *T. forsythia*, and *F. nucleatum*, respectively, surpassed 0.23%, 0.35%, and 2.94% of the total bacteria at baseline [78]. Furthermore, subgingival biofilm samples analyzed with qPCR indicated that unfavorable treatment outcomes occurred when >1000 cells/mL of *P. gingivalis*, *T. forsythia*, *A. actinomycetemcomitans*, *T. denticola*, and *P. intermedia* were detected at baseline [79]. All these results agree with those of the present work, which also highlighted the abundance of *P. gingivalis* at baseline as a negative predictor of NSPT.

### Limitations

Including additional periodontal pathogens was not feasible in this study. Thus, future studies should consider additional pathogens. Similarly, other biomarkers such as proinflammatory IL-1β, anti-inflammatory IL-10, and tumor necrosis factor-α could be included in future trials of both saliva and GCF. We also recommend monitoring the predictive potential of different biomarkers in this study over a longer period, up to 12 months, including both the active and maintenance phases. However, despite its limitations, this study underscores the value of using periodontal pathogens to obtain site-specific responses to treatment and provides novel insight into the potential of salivary GST and Annexin-1 to predict the outcomes of NSPT. This aspect of these biomarkers has been rarely investigated by previous studies. However, further trials are encouraged to validate the findings of this study before being adapted to clinical practice.

## 5. Conclusions

Increased levels of *P. gingivalis* in subgingival biofilm samples and an increase in PPD at baseline are negative site-specific predictors for outcomes of NSPT. Additionally, a higher rate of pocket closure could be predicted when treating the proximal sites of teeth. A high concentration of salivary MMP-9 is associated with poorer outcomes following NSPT at the whole oral cavity level, whereas increased salivary GST and Annexin-1 levels are positively associated with pocket closure after NSPT. These biomarkers could provide clues to develop more appropriate treatment plans such as surgical interventions and the use of adjunctive therapies as necessary.

## Figures and Tables

**Table 1:** Basic characteristics of the study population (n = 68).

Variables	
Age ^†^	55.4 ± 6.3
Sex ^‡^	
Male	31, 45.6%
Female	37, 54.4%
Stage ^‡^	
2	19, 27.9%
3	32, 47.1%
4	17, 25.0%
Grade ^‡^	
B	44, 64.7%
C	24, 35.3%
Periodontal pockets (4–6 mm)	
Total	1111, 100.0%
≥5 mm (no BOP) ^‡^	692, 62.3%
PPD ≥ 4 mm + BOP ^‡^	419, 37.7%
Missing teeth ^†^	4.2 ± 1.3

^†^ Mean ± SD. ^‡^ Frequency, percent.

**Table 2:** Clinical outcomes at the baseline and endpoint of the study.

	Baseline	Endpoint
PI ^†^	45.6 ± 8.2	11.2 ± 6.2 *
BOP ^†^	53.7 ± 8.2	13.1 ± 5.3 *
PPD ^†^	4.7 ± 0.7	3.8 ± 0.6 *
CAL ^†^	3.5 ± 0.6	3.23 ± 1.1 *
Whole-mouth outcomes ^‡^		
Success		42, 61.8%
Failure		26, 38.2%
Teeth outcomes ^‡^		
Maxillary (n = 465)		
Success		332, 71.4%
Failure		133, 28.6%
Mandibular (n = 364)		
Success		249, 68.4%
Failure		115, 31.6%
All teeth (n = 829)		
Success		581, 70.1%
Failure		248, 29.9%
Site outcomes (n = 1111) ^‡^		
Success		790, 71.1%
Failure		321, 28.9%
Residual pockets		
≥5 mm (no BOP) ^‡^		120, 10.8%
PPD ≥ 4 mm +BOP ^‡^		201, 18.1%

PI: plaque index; PPD: probing pocket depth; CAL: clinical attachment level; BOP: bleeding on probing. ^†^ Mean ± SD. ^‡^ Frequency, percent. * Significant difference at *p* < 0.05 using paired *t*-test.

**Table 3:** Microbiological outcomes at the baseline and endpoint of the study.

	Baseline	Endpoint	*p*-Value *
*P. gingivalis* ^†^			
+	250, 22.5%	187, 16.8%	
−	861, 77.5%	924, 83.2%	<0.001
*F. nucleatum* ^†^			
+	1053, 94.8%	524, 47.2%	
−	58, 5.2%	587, 52.8%	<0.001
Unresponsive sites ^†^			
*P. gingivalis* +		72, 38.5%	
*F. nucleatum* +		142, 27.1%	
Simultaneous presence of bacteria ^†^			
Total sites		98, 8.8%	
Unresponsive sites		98, 30.5%	

*P. gingivalis*: *Porphyromonas gingivalis*; *F. nucleatum*: *Fusobacterium nucleatum*. ^†^ Frequency, percentage. * Significant difference at *p* < 0.05, chi-square *t*-test.

**Table 4:** Logistic regression for predictors of successful site-specific and whole oral cavity outcomes.

Predictors	B	SE	*p*-Value	Exp (B)	95% CI for Exp (B)
Site-specific					
Tooth type ^a^					
Premolar	0.011	0.171	0.95	1.011	0.723 to 1.413
Molar	−0.194	0.202	0.33	1.214	0.817 to 1.803
Mandible ^b^	−0.130	0.142	0.34	0.878	0.665 to 1.160
*F. nucleatum*	0.015	0.310	0.96	1.015	0.552 to 1.865
*P. gingivalis*	−0.317	0.152	0.006	0.997	0.180 to 0.463
PPD	−0.457	0.118	<0.001	0.633	0.503 to 0.799
CAL	0.067	0.103	0.51	1.069	0.873 to 1.309
Site ^c^					
Distal	0.317	0.144	0.03	1.374	1.027 to 1.837
Facial	0.308	0.261	0.27	1.360	0.783 to 2.363
Oral	−0.657	0.416	0.11	0.519	0.229 to 1.172
Overall model accuracy	71.1%				
Whole-mouth ^†^					
MMP-9	−0.208	0.102	0.04	0.798	0.799 to 1.000
GST	0.896	0.364	0.01	2.449	1.252 to 5.308
Annexin-1	0.750	0.241	0.002	1.071	1.261 to 4.275
Overall model accuracy	79.4%				

PPD: probing pocket depth; CAL: clinical attachment level; *P. gingivalis*: *Porphyromonas gingivalis*; *F. nucleatum*: *Fusobacterium nucleatum*; B: regression coefficient; SE: standard error; Exp (B): odds ratio; CI: confidence interval; MMP-9: matrix metalloproteinase-9; GST: glutathione S-transferase. Reference: ^a^ anterior teeth; ^b^ maxilla; ^c^ mesial. ^†^ The model was adjusted for sex, age, and clinical parameters (plaque index, bleeding index, probing pocket depth, clinical attachment loss, stage, and grade). Bold font indicates a significant difference at *p* < 0.05.

## Data Availability

The data presented in this study are available from the corresponding author upon request due to legal and ethical reasons.
